# Molecular Characterization, Purification, and Mode of Action of *Enterocin* KAE01 from Lactic Acid Bacteria and Its In Silico Analysis against MDR/ESBL *Pseudomonas aeruginosa*

**DOI:** 10.3390/genes13122333

**Published:** 2022-12-10

**Authors:** Asma Bashir, Kashif Ali, Khair Bux, Neha Farid, Mitra Khaireabadi, Khwaja Ali Hassan, Abrar Hussain, Kiran Fatima, Shahab Mehmood, Syed Ali Haider, Ralf Herwig

**Affiliations:** 1 Department of Biosciences, Faculty of Life Sciences, Shaheed Zulfikar Ali Bhutto Institute of Science and Technology (SZABIST), Karachi 75600, Pakistan; 2 Department of Basic Science, Faculty of Biology, Hakim Sabzevari University, Sabzevar 96186-76115, Iran; 3 Molecular Biology & Structural Biochemistry Research Laboratory, Department of Biochemistry, University of Karachi, Karachi 75270, Pakistan; 4 Faculty of Life Sciences, Balochistan University of Information Technology, Engineering and Management Sciences, Quetta 87300, Pakistan; 5 Office of Research, Innovation and Commercialization (ORIC), Ziauddin University, Karachi 75600, Pakistan; 6 Laboratories PD Dr. R. Herwig, 80337 Munich, Germany

**Keywords:** probiotics, microbes, PCR, matrix proteins, bacteriocin, *Pseudomonas aeruginosa*

## Abstract

Bacteriocins are gaining immense importance in therapeutics since they show significant antibacterial potential. This study reports the bacteriocin KAE01 from *Enterococcus faecium,* along with its characterization, molecular modeling, and antibacterial potency, by targeting the matrix protein of *Pseudomonas aeruginosa*. The bacteriocin was purified by using ammonium sulfate precipitation and fast protein liquid chromatography (FPLC), and its molecular weight was estimated as 55 kDa by means of SDS-PAGE. The bacteriocin was found to show stability in a wide range of pH values (2.0–10.0) and temperatures (100 °C for 1 h and 121 °C for 15 min). Antimicrobial screening of the purified peptide against different strains of *P. aeruginosa* showed its significant antibacterial potential. Scanning electron microscopy of bacteriocin-induced bacterial cultures revealed significant changes in the cellular morphology of the pathogens. In silico molecular modeling of KAE01, followed by molecular docking of the matrix protein (qSA) of *P. aeruginosa* and KAE01, supported the antibacterial potency and SEM findings of this study.

## 1. Introduction

The recent rise in multi-drug-resistant bacteria is alarming, hence necessitating the discovery of novel antimicrobial drugs. Biotechnology has proved efficient in identifying and synthesizing various new antimicrobials to meet the rising need for medications against multi-drug resistance. Antibiotics are currently being replaced by these antibacterial compounds as treatments for many microbial illnesses and disorders. Multi-drug resistance (MDR) is associated with higher mortality rates and more expensive medical care and has a high impact on the effectiveness of currently available medications [1].

The pathogenic strain of the *Pseudomonadaceae* family, *P. aeruginosa*, is Gram-negative and has been found to be very hard to treat, as it has shown resistance against several regularly used antibiotics, such as Penicillin, Vancomycin, and Oxacillin [2]. Due to *P. aeruginosa’s* capacity to spread this trait to other pathogenic strains via the food chain, DNA fragments, the bacterial gene pool, or bacteriophages, it has become a severe threat to human health. Therefore, an alternative treatment is urgently required against this pathogen to cure illness without raising the possibility of the development of resistance against new medications [3].

Bacteriocins are protein-based compounds with antibacterial action that can limit the harmful activity of related and similar bacterial strains [4]. Prokaryotic organisms produce bacteriocins in order to increase their chances of survival against other germs in the immediate environment. Many research studies have focused on *Enterococcus* spp., which are among the bacterial strains capable of producing bacteriocins [5,6,7]. Lactic acid bacteria (LAB) are widespread producers of bacteriocins, which have broad-spectrum antibacterial activity. There are several other types of enterocins, but the first one to be fully defined was enterocin A, which is produced by some strains of *E. faecium* [8,9]. Class IIa bacteriocins, such as *Enterococcus faecium’s* enterocin P (EntP) and enterocin A (EntA), are antimicrobial peptides (AMPs) that kill bacteria. Apart from Class IIa bacteriocins, there are four classes of enterocins: Class I enterocins (lantibiotics enterocins), Class II enterocins (small, non-lantibiotic peptides), Class III enterocins (cyclic enterocins), and Class IV enterocins (large proteins) [8]. There are approximately 71 amino acids encoded by the EntP structural gene and 44 encoded by the EntA gene. Although the EntP gene seems to be widely distributed in *E. faecium* strains of varying origins, EntP and EntA are bacteriocins with a broad antimicrobial spectrum that inhibits various pathogens. Because of its effectiveness against such a wide variety of microorganisms, EntA may find use as a natural antibacterial addition in the food sector [10]. Methicillin-resistant *Staphylococcus aureus* (MRSA) and *Pseudomonas* are pathogenic strains, and illnesses caused by these strains can be treated using bacteriocins generated by *Enterococcus* spp. Bacteriocins are a possible contender for many biotechnological applications, as they select their target microorganisms with extreme specificity [11].

The rational design of peptide-derived therapeutics usually requires structural characterization of the underlying protein–peptide interaction. Protein–peptide docking methods can be divided into three categories: template-based docking; local docking; and global docking. Different approaches offer different levels of prediction accuracy, often determined by the amount of interaction information provided as input. The available methods use different ways of defining the binding site. RoseTTa FlexPepDock [12], DynaDock [13], and PepCrawler [14] require an initial model of the complex prepared by the user. By contrast, HADDOCK [15] can automatically place the peptide in the proximity of the binding site defined by a user-provided list of interface residues [16]. The applicability of these methods, i.e., AutoDock Vina [17], Gold [18], or Surflex-Dock [19], is limited to short peptides (up to a few amino acids). Finally, local docking methods can be used to refine medium-quality models to a better resolution, e.g., PIPER-FlexPepDock [20].

As the number of infectious diseases is rising, bacteriocins are promising options for the treatment of pathogens [21,22]. Bacteriocins have also been utilized for medicinal and human health applications [23,24]. Their effectiveness against clinically significant pathogens demonstrates the therapeutic potential of these bacteriocins against bacterial infections [25]. Specifically, bacteriocins have a number of advantageous features that make them excellent alternatives to antibiotics [26]. Very recent studies [27,28,29,30] that reported on bacteriocins have been very specific. Either they reported only the identification or characterization and structural identification or just applications [31,32,33].

To the best of our knowledge, this is one of the very few comprehensive studies to report the identification and characterization of a specific bacteriocin, along with its structural modeling and applications. This study aimed to identify, purify, and characterize the enterocin and identify the enterocin genes EntA and EntP. The evaluation of the mode of action of the enterocin (KAE01) by means of modeling and exploring its binding to the matrix protein through molecular docking simulations was also targeted in the presented study. Therefore, this study could pave the way for designing potential antimicrobial peptides against multi-drug-resistant bacterial pathogens.

## 2. Materials and Methods

### 2.1. Bacterial Strains and Culture Conditions

The isolation of lactic acid bacteria (LAB) was performed using diverse sources, including dairy products (fresh yogurt, milk, and cheese) and vegetables (tomatoes and cucumber), bought from the local market. Fresh yogurt and milk samples were directly swabbed on MRS media, whereas cheese, tomatoes, and cucumber paste were swabbed on the respective media. Collected bacterial samples were inoculated into MRS broth medium and incubated at 37 °C for 24 h in an aerobic environment. The resulting bacterial suspensions were streaked on the selective bile esculin agar medium for *Enterococcus* species and incubated for 24 h at 37 °C [1,2].

### 2.2. Identification of Enterococcus *spp.*

The *Enterococcus* bacterial strains used were grown in the presence of 4% bile (*v*/*v*). These bacterial cells hydrolyze esculin to esculetin, which reacts with ferrous ions (Fe^3+^) and produces dark-brown to black precipitates on the agar plate. Black colonies of *Enterococcus* bacteria named KAE01 grew on the media. The identification and characterization of *Enterococcus* bacteria were performed using microscopic examination through Gram staining and biochemical testing [1,14].

### 2.3. Genomic DNA Extraction

Fresh *Enterococcus* culture was used for the extraction of DNA using the EZ-10 Spin Column Bacterial Genomic DNA Miniprep Kit (Bio Basic, Markham, Canada). DNA integrity and concentration were determined by taking optical density measurements using a NanoDrop spectrophotometer (ThermoFisher Scientific, Waltham, USA) [34].

### 2.4. Amplification and Sequencing of 16S rRNA Gene Using PCR

The amplification of the desired DNA sequence was carried out using PCR with universal primer sets: forward primer 3′-AGAGTTTGATCCTGGCTCAG-5′ and reverse primer 5′-GGCTGCTGGCACGTAGTTAG-3′. The thermal cycler was programmed with a pre-incubation period of 2 min at 94 °C and then 35 cycles, each consisting of denaturation for 30 s at 94 °C, annealing for 30 s at 55 °C, and elongation for 1 min at 72 °C. A final elongation of 10 min at 72 °C followed the 35 cycles. The amplicons were visualized through 1% agarose gel electrophoresis with reference to a 1 kbp DNA ladder. The PCR product was quantified using a NanoDrop spectrophotometer and purified using the QIAquick purification kit (Qiagen, Germantown, USA. The purified amplicons were then sent to Molecular Products Co. Pakistan for sequencing [35].

### 2.5. Phylogenetic Analysis

The sequences were edited using Bioedit V.5.0.9 [36] to perform the phylogenetic analysis. For the identification of the nearest neighbors, a BLAST search was conducted using the NCBI genome database server (http://blast.ncbi.nlm.nih.gov/Blast.cgi (accessed on 3 August 2021) [37]. Alignment, phylogenetic, and molecular evolutionary analyses were conducted using MEGA version 5.0 [38,39]. To confirm the reliability of the phylogenetic tree, a bootstrap test and reconstruction were performed 1000 times [40].

### 2.6. PCR Screening for Enterocin Structural Genes

Genes encoding for enterocin structural proteins were investigated using the PCR amplification technique. Specific primers for EntP: forward primer 3′-AGAGTTTGATCCTGGCTCAG-5′ and reverse primer 5′-GGCTGCTGGCACGTAGTTAG-3′; for EntA: forward primer 3′-AAAATAAATGTACGGTCGATTGG-5′ and reverse primer 5′-CCAGCAGTTCTTCCAATTTCA-3′. PCR product size and integrity were determined through agarose gel electrophoresis (1%), using a DNA ladder of 1 kb as a reference (Cat. no. 10787018) [41].

### 2.7. Extraction and Purification of Enterocin KAE01

The culture of *E. faecium* KAE01 was grown in 2 L of MRS medium for 48 h at 37 °C. The supernatant was separated by centrifugation and adjusted to pH 7.0, followed by filtration with a 0.45μm filter. The filtrate was then subjected to ammonium sulfate precipitation at 40% and 60% saturation, and the resultant pellet was resuspended in potassium phosphate buffer (pH 7.0) and then dialyzed for 12 h at 4 °C using a 2 kDa cut-off membrane. The crude bacteriocin solution underwent cation-exchange chromatography (Sartobind^®^ Membrane Adsorber Unit, Matrix S-type exchanger, S15F, Darmstadt, Germany) for further purification. The pumps were primed as A and B with filtered buffer A (10 mM Tris–HCl, pH 7.0) and B (10 mM Tris–HCl, pH 7.0, 1 M NaCl), respectively. The Mono S column was equilibrated (1 mL volume) with 5 volumes of buffer A and 10 volumes of buffer B and then with 5 volumes of buffer A. The sample (20 mL-5 mg/mL) was loaded and the peaks were monitored at a wavelength of 210 nm, and fractions (2 mL each) were collected.

### 2.8. Molecular Mass Determination by Tricine SDS–PAGE

The molecular mass of the enterocin was determined by running the purified enterocin KAE01 preparation on Tricine SDS-PAGE using Schagger and von Jagow’s proposed protocol with slight modifications [42]. A mixture of 25 μL of the sample and 25 μL of buffer (Tris base 100 mM, glycine 100 mM, SDS 4%, and bromophenol blue 0.01%) was heated for 5 min at 100 °C and run on a vertical gel at 30 V for 60 min and then at 100 V for 4 h. The gel was stained using coomassie brilliant blue staining, and a protein ladder with sizes from 10 to 100 kDa was used to determine the molecular mass. The concentration of the protein was calculated by using ImageJ software: http://rsbweb.nih.gov/ij/download.html (accessed on 21 October 2022) [43].

### 2.9. Antimicrobial Activity Assay

After purification, the antimicrobial activity of the purified fraction was assessed using the AWDA (agar well diffusion assay) method. This method was performed using 0.8% Mueller–Hinton agar medium plates inoculated with three strains of *P. aeruginosa* as indicator organisms. Two strains of *P. aeruginosa*, i.e., PA01 and PA03, were used as clinical strains, whereas PA02 was the standard strain from ATCC BAA-1744. This soft Mueller–Hinton agar medium was overlayed on the base Mueller–Hinton agar plates. Then, 100µL of bacteriocin was inserted into wells of 6 mm diameter cut out on prepared agar plates, and then the culture was incubated overnight. After incubation, a zone of inhibition was formed because of the antimicrobial properties of the bacteriocin, and the inhibition zone diameter of microbial growth was estimated [41]. The bacteriocin was tested in the concentration range of 25 µg/mL to 200 µg/mL for the determination of the Minimum Inhibitory Concentration (MIC).

### 2.10. Influence of pH, Heat, and Proteolytic Enzymes on Enterocin KAE01

#### 2.10.1. Temperature

Purified enterocin, redissolved in 0.05% acetic acid, was tested at various temperatures (20 °C, 4 °C, 30 °C, 60 °C, and 100 °C) for 1 h, as well as under autoclave conditions (121 °C at 15 psi for 15 min) to see how enterocins were affected by the temperature. The residual bacteriocin activity against the bacterial indicator strains was measured by the agar well diffusion method [44].

#### 2.10.2. pH

Purified enterocin was redissolved in 0.05% acetic acid, which was adjusted to different pH levels of 2.0, 3.0, 4.0, 5.0, 6.0, 7.0, 8.0, 9.0, and 10.0 with 1.0 M HCl or 1.0 M NaOH to test pH stability. For one hour, samples were incubated at 37 °C. By using the agar well diffusion method against the bacterial indicator strains, the residual bacteriocin activity was assessed [44].

#### 2.10.3. Enzymes

Different enzymes were added to purified enterocin, redissolved in 0.05% acetic acid, to test its enzymatic sensitivity at 37 °C: proteinase K (pH 7.5), trypsin (pH 8.0), pepsin (pH 2.0), and chymotrypsin (pH 3.0). The enterocin enzyme mixture was incubated for 1 h and then boiled for 5 min to inactivate the enzymes. The enzymes were used at a concentration of 75 µg/mL. The agar well diffusion test was used to determine the remaining antibacterial activity against the bacterial indicator strains [44].

### 2.11. Statistical Analysis

Data were analyzed using SPSS version 25. The Mean and Standard Deviation were computed for numeric variables. ANOVA was applied to compare the zone diameters with respect to the pH level, temperature, and proteolytic enzymes. A *p*-value ≤ 0.05 was considered statistically significant.

### 2.12. Scanning Electron Microscopy (SEM)

The indicator strains of *P. aeruginosa* were exposed to enterocin KAE01 and investigated by SEM. The cultures of the indicator strains were treated with enterocin KAE01 in their exponential phase (optical density of 0.6 at 600 nm) and incubated for 2 h at 37 °C. The control groups were grown without enterocin. The treated and control samples were harvested, washed with potassium phosphate buffer, and fixed with 2.5% (*v*/*v*) glutaraldehyde at 4 °C for 4 h. The samples were then dehydrated with gradient alcohol solutions (30%, 50%, 70%, 85%, 90%, and 100%). After lyophilization and gold coating, the samples were observed by scanning electron microscopy (S4800, Hitachi, Japan) [45].

### 2.13. Ab Initio Modeling of KAE01

The 3D model of the peptides that formed KAE01 EntP was generated using Robetta with RoseTTa fold as the default option. It uses a deep learning modeling method to build the model and the AB method for the target sequence model, which are non-homologous in PDB (https://robetta.bakerlab.org (accessed on 15 June 2022). Further, the quality of the generated models was evaluated using the online servers of QMEAN (https://swissmodel.expasy.org/qmean (accessed on 17 June 2022) [46] and Ramachandran plots (https://zlab.umassmed.edu/bu/rama/ (accessed on 20 June 2022) [47].

### 2.14. Peptide–Protein Docking

#### 2.14.1. Protein Preparation

The three-dimensional (3D) structure of the matrix protein of *P. aeruginosa* A (UniprotKB ID: Q9l4×3) was modeled using MODELLER (v 9.23) software. Two templates with maximum sequence identity and query coverage were used for better homologous structure prediction (Template 1: PDB ID = 5OE3, query coverage = 77%, sequence identity = 100%; Template 2: PDB ID = 1ULT, query coverage = 96%, sequence identity = 25%). Model refinement was performed using the Galaxy Web Server, which relaxes the structure using molecular dynamic simulations (https://galaxy.seoklab.org (accessed on 20 July 2022) [29,48]. Different online servers were used for the structure assessment, including Ramachandran plots (https://zlab.umassmed.edu/bu/rama/ (accessed on 25 July 2022) and QMEAN (https://swissmodel.expasy.org/qmean (accessed on27 July 2022).

#### 2.14.2. Peptide–Protein Docking

The simulation of molecular docking was performed on the ClusPro protein–protein docking server (https://cluspro.bu.edu (accessed on 28 July 2022) [49,50,51,52]. ClusPro 2.0 follows three computational steps: (i) rigid-body docking using the fast Fourier transform (FFT) correlation approach, (ii) root-mean-square deviation (RMSD)-based clustering of the structures generated to find the largest cluster that will represent the likely models of the complex, and (iii) refinement of selected structures. The docking results were visualized in PyMOL to confirm the binding positions of peptides and receptors. The predicted binding affinity (ΔG) and dissociation constant (KD) values were obtained from the Prodigy server (https://wenmr.science.uu.nl/prodigy/ (accessed on 31 August 2022).

## 3. Results

### 3.1. Isolation, Identification, and Phylogenetic Analysis of Enterococcus Faecium KAE01

**Figure 1:**
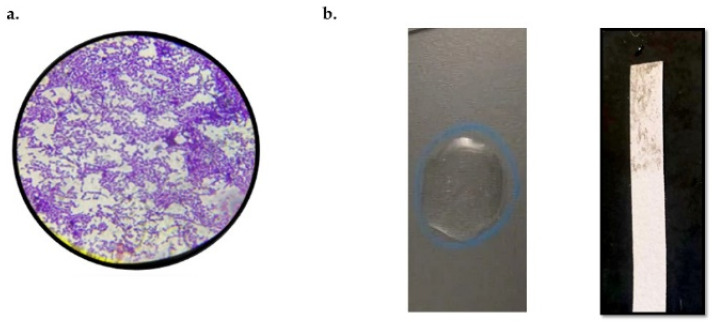
(**a**) Purple-colored cocci colonies observed after Gram staining of *Enterococcus faecium* KAE01. (**b**) Biochemical tests show that strain KAE01 is negative for catalase and oxidase, as no bubbles are formed and no purple stain is seen on the strip, respectively.

**Figure 2:**
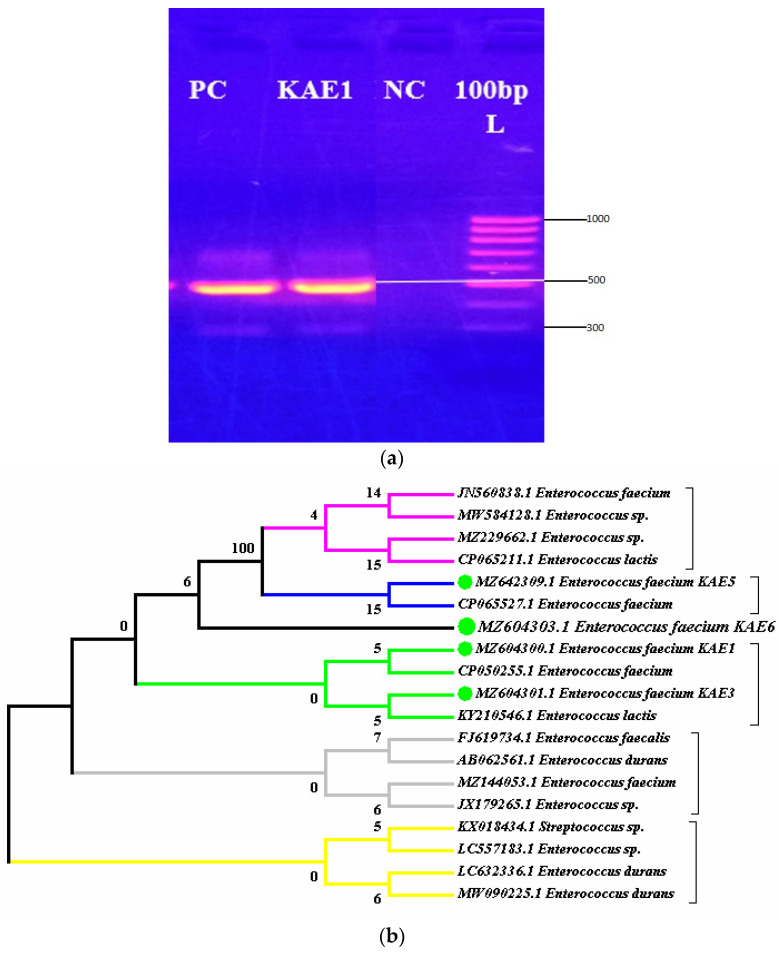
(**a**) Agarose gel electrophoresis showing Positive Control (Lane 1), 16s rDNA fragment amplified by PCR from genomic DNA of strain KAE01, corresponding to 500 bp (Lane 02), Negative Control (Lane 3), and DNA molecular weight marker of 1000 bp (Lane 4). (**b**) Phylogenetic tree constructed based on 16s rDNA sequence amplified from genomic DNA of strain *E. faecium* KAE01 and other strains obtained from BLAST results.

Strain KAE01, isolated from yogurt and identified using morphological, biochemical, and molecular techniques, was selected for the study. Strain KAE01 is Gram-positive, catalase- and oxidase-negative, and rod-shaped (Figure 1). It was also able to produce ammonia from arginine and ferment different carbohydrates, such as galactose, raffinose, fructose, dextrose, maltose, mannose, and sucrose. Based on these tests, strain KAE01 was found to belong to the genus *Enterococcus* [32]. Species-level identification was performed using 16S rDNA amplification and sequencing. The size of the amplified PCR product was found to be approximately 500 bp (Figure 2a). The PCR product was sequenced and used for a homology search for 16S rDNA sequences available in the database using BLAST (NCBI). The phylogenetic analysis of sequences closer to the sequence obtained based on the BLAST result showed 99.9% similarity to *Enterococcus faecium* NRIC 0677, as shown in Figure 2b. The sequence was submitted to GenBank (NCBI) with the accession number MZ604300.1.

### 3.2. Analysis of Enterocin Structural Genes

**Figure 3:**
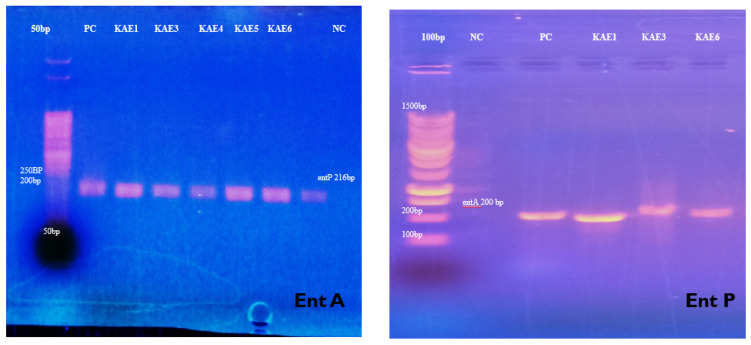
Agarose gel electrophoresis of EntA and EntP genes showing bands at 200 bp and 216 bp, respectively.

Enterocin KAE01 genes were detected after the PCR amplification of genomic DNA from *E. faecium* KAE01 using primers that targeted two known enterocin genes, EntP (216 bp) and EntA (200 bp) (Figure 3). A homology score of 91% was found between the gene sequence and *E. faecium* FSIS1608820 (GenBank identifier CP028727.1). The purified enterocin KAE01 had gene sequences of *E. faecium* KAE 01 EntA (consensus sequence F/R), which is GAGGAAGTAGAAAATGAAAAAAGCAAAATTGATACTTAGTACAATCGTCTGACATCTTTATGTAGTGGTTTTTTAGGGACTGGGACGGTGGATGCCAGTGAATATAATAATAAAGTTGTTTCGCTTCGAGCTGCTAGTGATACAACGCTTACTGTGTCACGCATACCTCATAATCCTATTCTATATTTTGAAATTTCAATGGAAAATTATAATCAATCTGACTCAACCCAACGTTGGGTAATGGAG, and of *E. faecium* KAE 01 EntP (consensus sequence F/R), which is ATGAACCCGCTGAGCCCGAGCGCGTTTACCTTTTGCCCGCTGGGCTTTATTCCGCTGGGCTTTCTGGAAATTTGGCGCAACTGCTGGGGCATGCATCATGTGAAATTTCATGGCGGCAAATATTATGGCAACGGCGTGTATTGCACCAAACAGGGCTGCAGCGTGAACTGGGGCCAGGCGAACGTGAGCGCGATTCAGGAACGCCTGAGCCGAACAACAGCCCGCGCATTCGCTATGGCTTTAGCCCGATGGGCGAAGTGCAGAGCGAAATTGGCTAA.

### 3.3. Purification of Enterocin KAE01

To obtain a partially pure product, a two-step simple separation process for enterocin purification was applied. The protein content of the supernatant of the *E. faecium* culture was subjected to various concentrations of ammonium sulfate, i.e., ranging from 10% to 80%. The total protein content with the highest antimicrobial activity was found to be saturated at 60% ammonium sulfate. As a result, in subsequent experiments, 60% ammonium sulfate was used. 

**Figure 4:**
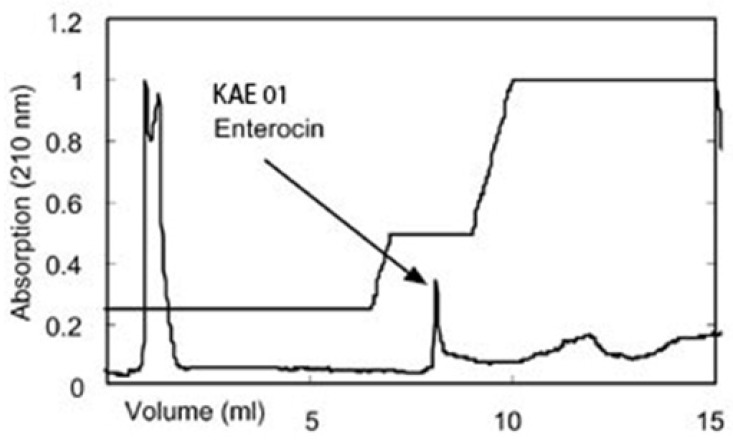
Purification to homogeneity of enterocin protein using fast-performance liquid chromatography of KAE01.

The active sample was purified in the second step using cation-exchange chromatography. There was one peak among all purified enterocins (Figure 4) that had antibacterial activity. The Fast Flow active fraction was purified further using Sephadex G-25 and tested for antibacterial activity.

### 3.4. Molecular Mass Determination by Tricine SDS–PAGE

**Figure 5:**
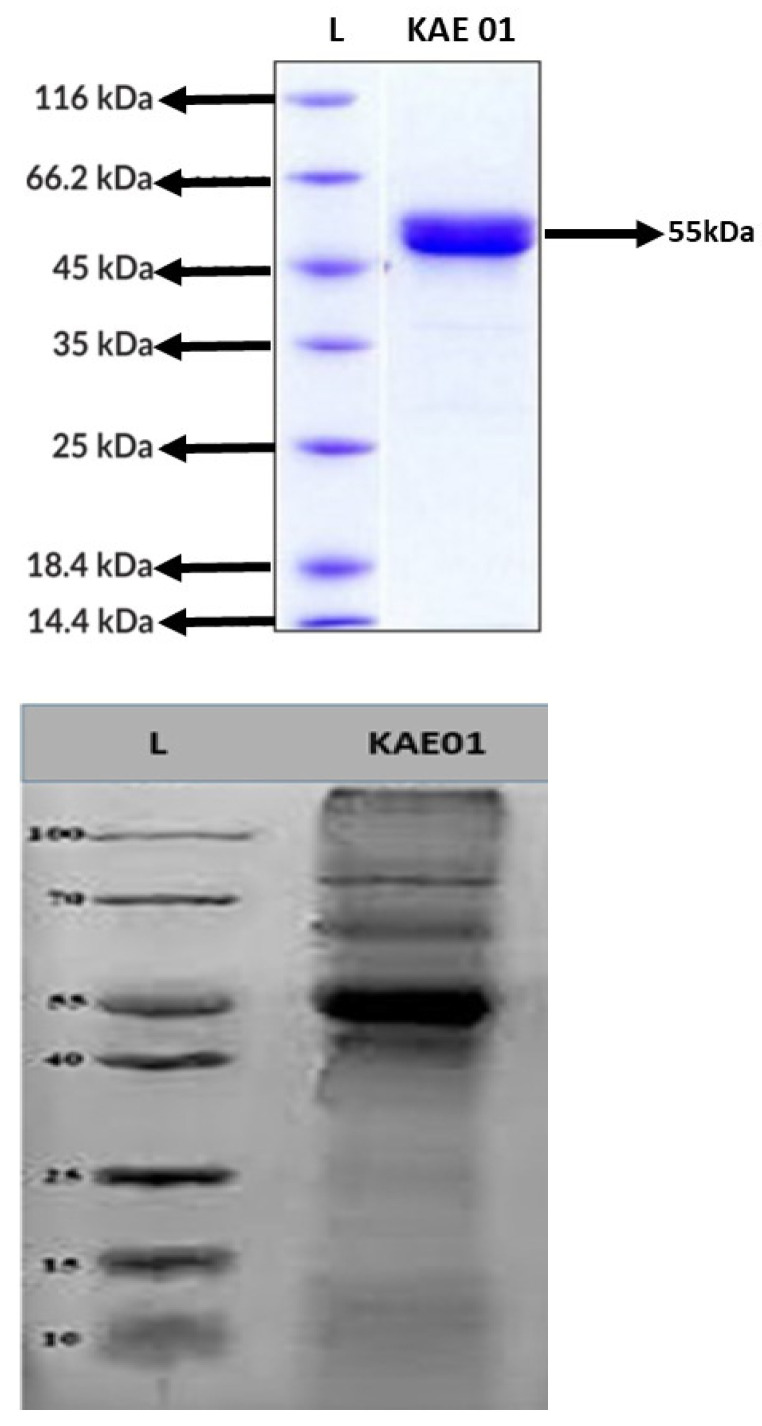
Identification of molecular weight of purified enterocin KAE01 using SDS-PAGE. Lane 1: standard molecular size ladder; Lane 2: purified enterocin KAE01 with approximate size of 55 kDA.

The Tricine SDS-PAGE results revealed a peptide band of 55 kDa (Figure 5). The *P. aeruginosa* strains, i.e., PA01, PA02, and PA03, were inhibited by the enterocin KAE01 band, which was found to be consistent with the SDS-PAGE findings and indicated that a pure protein sample had been obtained. By using the active band from the SDS-PAGE gel, it was recognized as a bacteriocin because of the molecular weight of 55 kDa (5 mg/mL using ImageJ software: http://rsbweb.nih.gov/ij/download.html (accessed on 21 October 2022) with reference to the standard protein ladder ranging from 10 to 100 kDa using SDS-PAGE electrophoresis. Based on the identified sequences of the EntA and EntP genes, the amino acid sequences of the corresponding proteins were predicted as LANFALHFAHRAKAIANARAVVRAQAFLNRAHVRLAPVHAAALFGAIHAVAIIFAAMKFHMMHAPAVAPNFQKAQRNKAQRAKGKRARAQRVH for EntP and MKKAKLILSTVLTSLCSGFLGTGTVDASEYNNKVVSLRAASDTTLTVSRIPHNPILYFEISMENYNQSDSTQRWVME for EntA.

### 3.5. Antimicrobial Activity of Purified Enterocin KAE01

Purified enterocin KAE01 showed activity against Gram-negative indicator microorganisms, i.e., MDR/ESBL *P. aeruginosa* strains PA01, PA02, and PA03, as shown in Table 1. Additionally, 100µg/mL of enterocin KAE01 proved to be the MIC value and was sufficient to halt *P. aeruginosa* growth. This result is in line with what Class IV enterocin bacteriocins are known for [53].

### 3.6. Influence of pH, Heat, and Proteolytic Enzymes on Enterocin KAE01

The Cell-Free Supernatant (CFS) and purified enterocin KAE01 maintained antibacterial activity over a wide pH range from 2 to 10. The antimicrobial activity of the bacteriocin was stabilized by up to 25% under these conditions; the antibacterial activity of the enterocin remained steady at 100 °C for 60 min. The enterocin, however, lost 25% and 50% of its activity after 15 min of heating at 121 °C. After being treated with trypsin, pepsin, proteinase K, and chymotrypsin, the purified enterocin reduced its antibacterial activity against MDR/ESBL *P. aeruginosa* PA01 (Table 2, Table 3 and Table 4). The antimicrobial activity was measured in triplicate. There were statistically significant differences in the zone-of-inhibition diameters with respect to the pH level, temperature, and proteolytic enzymes (*p* < 0.05).

### 3.7. Scanning Electron Microscopy (SEM) Analysis

**Figure 6:**
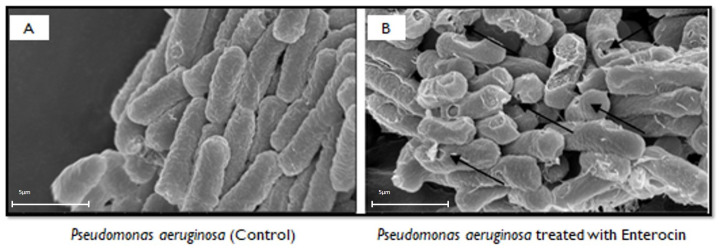
Mode of action of enterocin KAE 01 using SEM; *P. aeruginosa* (**A**) control and (**B**) treated. Arrows point toward cells that have ruptured due to antimicrobial effect of enterocin (Scale: 3 kV; X60000).

According to SEM analysis, without the enterocin KAE01 treatment, control *P. aeruginosa* cells had a smooth surface and a full appearance, while a considerable change in the cell shape and the appearance of holes and wrinkles on the cell surface after the enterocin KAE01 treatment (Figure 6) indicate that enterocin KAE01 directly affected the cell wall. The enterocin KAE01 treatment for two hours caused the cell wall to develop pores, and the cells seemed to be plasmolyzed. These results demonstrate that cell membrane disruption and subsequent cell lysis are connected to the bactericidal activity of enterocin KAE01. Based on these findings, enterocin KAE01′s mode of action is most similar to that of Class IV enterocins.

### 3.8. Peptide–Protein Docking

**Figure 7:**
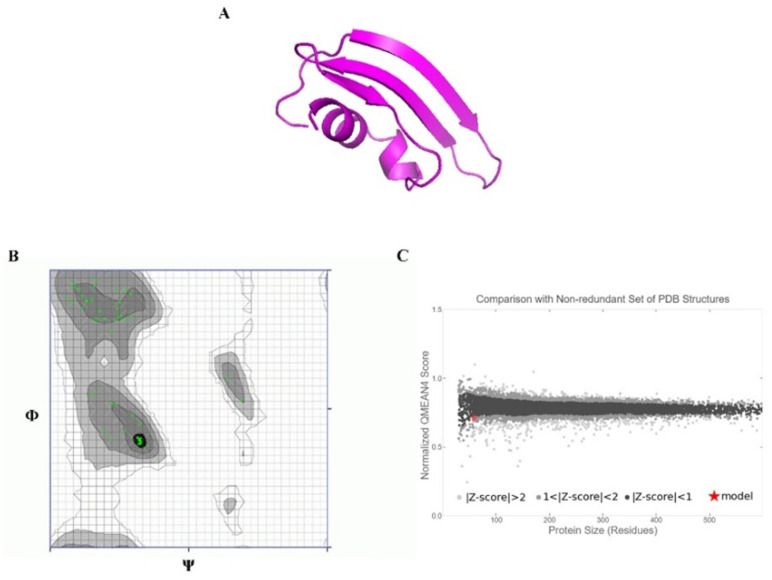
KAE01 EntP model obtained from AB method: (**A**) ribbon view of 3 KAE01 EntP model, (**B**) Ramachandran plot, and (**C**) qmean Z-score plot.

**Figure 8:**
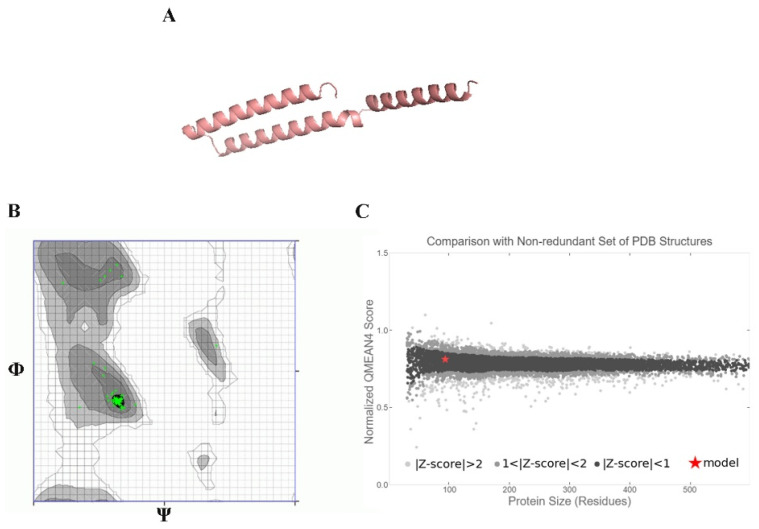
KAE01 EntA model: (**A**) ribbon view of 3 KAE01 EntA model, (**B**) Ramachandran plot, and (**C**) qmean Z-score plot.

**Figure 9:**
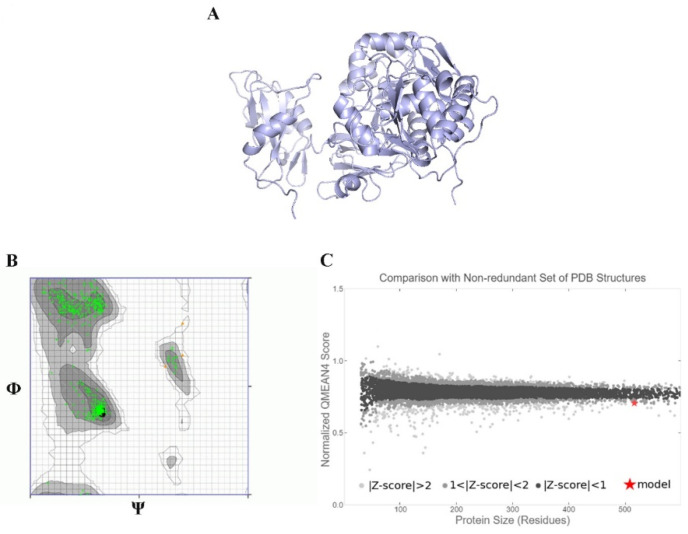
Matrix protein model: (**A**) ribbon view of matrix protein model, (**B**) Ramachandran plot, and (**C**) qmean Z-score plot.

To understand how antibacterial peptides exert their effects, peptide models (EntA and EntP) were developed for docking with the matrix protein of *P. aeruginosa*. The peptides were modeled with an ab initio method because there was no homologous protein in PDB to be used as a template. Two methods were used to obtain peptide models in the Robetta server: RoseTTa fold as the default option, which uses a deep learning modeling method to build the model, and the AB method to build a model for target sequences that are not homologous in PDB. Two different models were built with two RoseTTa fold and AB methods on the Robetta server for KAE01. The Ramachandran plot, qmean factor, and prosa z-score for all obtained models indicate good agreement between the model and experimental structures (Figure 7, Figure 8 and Figure 9).

The matrix protein of *P. aeruginosa* is a target of bacteriocins. In the first step, because there is no 3D structure of the matrix protein available for *P. aeruginosa*, comparative modeling using 5OE3 (PqsA N-terminal domain) from *P. aeruginosa* PAO1 and 1ULT from *Thermus thermophilus* using MODELLER v9.23 software was performed [54]. The matrix protein of the *P. aeruginosa* model has a chain with two separated domains that are connected with a short flexible linker. The N-terminal domain from 1 to 390 residues can be subdivided into three subdomains: two β-sheets connected by an internal two-fold symmetry surrounded by α-helices and a distorted β-sheet and a C-terminal domain from 400 to 500 residues. The Ramachandran plot analysis after structural refinement was performed to assess the quality of the predicted structure. Most residues (99.31%) of the structure were grouped in the most favored region, whereas only 0.68% of the residues lay in the outlier region, which indicates the good quality of the structure. The Ramachandran plot and a qmean of 0.63 confirmed that the model has good agreement with experimental structures (Figure 7). According to a previous report [54], this protein has highly conserved residues when compared with other aryl-CoA ligases, such as Gly307, Gly302, Ala278, Gly279, His308, and Tyr211 located in the N-terminal domain. The best binding mode of KAE01 EntP in the matrix protein complex of *P. aeruginosa* in two clusters with 49 members from 1000 bootstraps has a binding energy of −16.4 Kcal/mol and an inhibition constant of 0.89 pM.

**Figure 10:**
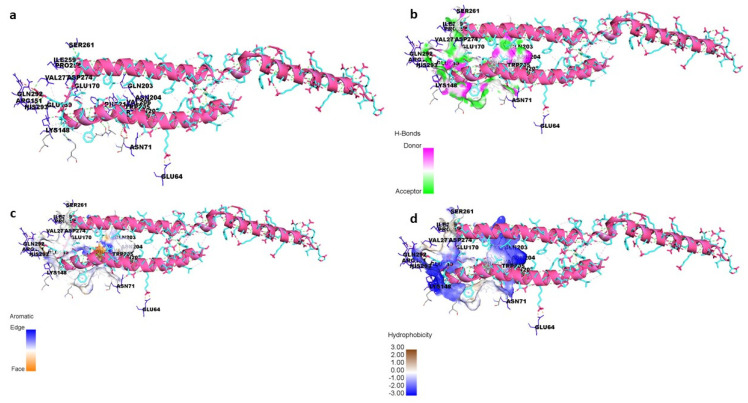
(**a**) Mode of binding of EntA (red) peptide with protein stabilized by non-bonding interactions with amino acid residues. (**b**) Hydrogen-bonding interactions of peptide with protein residues. (**c**) Representation of π–π interaction stabilizing the binding of EntA. (**d**) Hydrophobic cavity supporting peptide binding to protein.

EntA was observed to exhibit active binding since some amino acid residues, such as SER261, GLN292, ILE259, ASP274, ARG151, and LYS148, were found to stabilize peptide binding through hydrogen-bonding interactions (Figure 10a,b). In addition, GLU64, ASN71, GLN203, VAL277, GLU170, and ASN204 were observed to interact through π–π and hydrophobic interactions, as shown in (Figure 10c,d).

**Figure 11:**
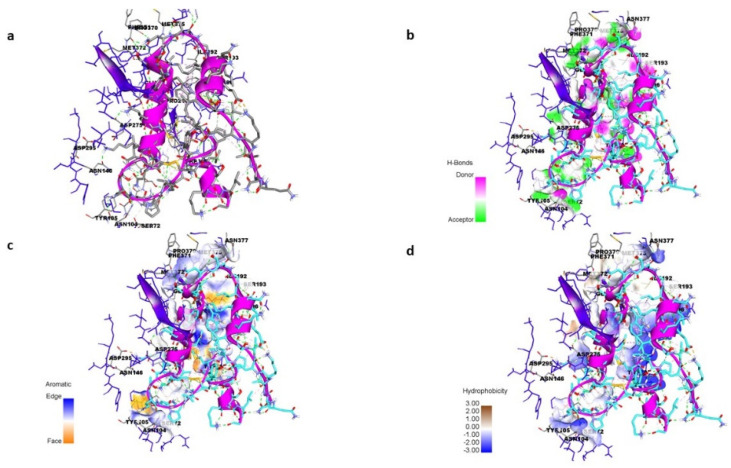
(**a**) Mode of binding of EntP (indigo) peptide with protein stabilized by non-bonding interactions with amino acid residues. (**b**) Hydrogen-bonding interactions of peptide with protein residues. (**c**) Representation of π–π interaction stabilizing the binding of EntP. (**d**) Hydrophobic cavity supporting peptide binding to protein.

Similarly, the binding of EntP was also found to be stabilized by different types of interactions with protein amino acid residues such as ASP275, ASP295, ASN195, TYR105, and MET372, which were observed to form hydrogen-bonding interactions with the peptide. Moreover, ASN 377, PRO370, SER193, PHE 371, MET 372, and ILE 193 were found to interact through *π–π* and hydrophobic interactions (Figure 11).

## 4. Discussion

Bacteriocins have the potential to be used in a variety of applications because it has been demonstrated that it is possible to create vast quantities of them without the use of pathogens. The versatility of bacteriocins allows for a wide range of applications, and their modular design allows for the creation of custom bacteriocins. The availability of bacteriocins broadens the options even more, enabling their usage in the home or workplace to identify bacteria in a variety of samples. In this research, *Enterococcus faecium* was isolated from a sample of commercial dairy products. The bacterial strain of *E.s faecium* KAE01 was identified using phylogenetic studies of its 16S rRNA gene sequence. The bacterial culture was therefore given the name *E. faecium* KAE01 (MZ604300.1), as genotypic procedures such as 16S rRNA gene sequencing are typically more accurate than phenotypic tests [41,55]. The bacterium is Gram-positive, facultative, catalase- and oxidase-negative, non-motile, and nitrate-reductase-negative and displays the normal phenotypic traits of an *Enterococcus* species.

In the current investigation, agar well diffusion assays revealed broad-spectrum antibiotic efficacy against MDR/ESBL bacteria, i.e., *P. aeruginosa* (PA01, PA02, and PA03). The strain of *E. faecium* produced an inhibitory agent, which was heat stable. As per the findings of this research, the CFS of the *E. faecium* KAE01 strain maintained its activity even when heated to 121 °C. The results of this investigation indicate that *E. faecium* produces the inhibiting substance(s) generated by *E. faecium* strain KAE01 and that it is bacteriocin-like. Some bacteriocins are known to not be released as bioactive peptides and need further extracellular processing by a general or particular enzyme; they have protease links between asp and pro that are effortlessly hydrolyzed in acidic conditions, releasing the bacteriocin in its active form [56]. The latter finding offers a potential explanation for the earlier finding that the highest antibacterial activity is seen in an acidic environment. Before being used, enterocin KAE01’s safety should be confirmed through additional tests, such as a hemolytic experiment. In the range of acidic pH values, enterocin was active, similar to nisin, which has a potent antibacterial effect at low pH levels before becoming inactive around pH 7–9. As a result, acidic environments are where nisin and isolated enterocin KAE01 were most active. Because enterocin KAE01 was stable in the low-pH range, acidic foods may be utilized to prepare it.

The activity was reduced when purified enterocin KAE01 FPLC fractions were exposed to proteases such as pepsin, trypsin, chymotrypsin, and proteinase K. Circular bacteriocins (head-to-tail-ligated cyclic antimicrobial peptides), including circularin A, Microcin J25, gassercin A, and AS-48, have been found to be extremely resistant to being broken down by some proteases [57,58]. The three-dimensional shape of these circular peptides may make recognition sites inaccessible, rather than the lack of enzyme digestion sites being the cause of this resistance. Circular peptides may become less vulnerable to the digestive enzymes produced by the target bacteria as a result of protease digestion, broadening the range of their activity.

SEM analysis revealed that enterocin KAE01 had destroyed PA01′s cell membrane, causing the cytoplasm to leak. It was therefore hypothesized that enterocins caused the cytoplasm to leak by rupturing the cell membrane, resulting in bacterial mortality. A number of physiochemical indicators associated with the cell membrane need to be investigated in order to further confirm the antibacterial activity pattern of this enterocin. According to the findings, enterocin damaged bacterial cell membranes and caused electrolytes to leak out, which results in bacterial cell membrane damage. The antibacterial mechanism of the peptide produced by *Enterococcus fecalis* isolated from Tibetan kefir against *P. aeruginosa* was also well explained by a study conducted by [59].

Two bacteriocin structural genes, EntA and EntP, were found on the chromosome of *E. faecium* KAE01 by polymerase chain reaction. This suggests that *E. faecium* KAE01 has the potential to produce multiple bacteriocins. It is well known that some bacteriocins that were initially believed to be large complex molecules or have higher molecular weights were further purified as smaller peptides [4,60]. This is true even though the detected bacteriocin structural genes encode low-molecular-weight peptides, and SDS-PAGE detected a high-molecular-weight peptide of 55 kDa [61]. This is a typical observation for Class II and Class IIb bacteriocins and Class IV enterocins [8,61,62,63,64,65]. Class IIa bacteriocins are linked to all of the bacteriocin structural genes discovered in this investigation. These bacteriocins, which are non-lantibiotic, are further split into three classes. Class IIa bacteriocins resemble pediocins and have potent antilisterial activities. The mature peptides of Class IIa bacteriocins have a conserved N-terminal YGNGVXC consensus sequence. Class IIb bacteriocins are heterodimeric and comprise two peptides. Individual peptides produce little to no antimicrobial activity, and the presence of both is necessary for full antimicrobial activity [62]. This organism’s acidocin bacteriocin was extracted as a single peptide with a molecular weight of 5400 and a predicted amino acid number of 50, and it showed bactericidal action [66]. According to [67,68], acidocin is a Class IIa bacteriocin that can form oligomers that can dissociate into monomers with increased activity. These monomers are maintained by the 45 kDa membrane [69] Class IIb bacteriocins and are all encoded by the structural genes EntB, EntP, and EntA.

Although it has long been believed that bacteriocins from Gram-positive bacteria do not hinder the growth of Gram-negative bacteria, there have been more and more instances of this assumption being violated. This investigation showed that the yogurt-isolated *E. faecium* KAE01 strain inhibited the growth of three MDR/ESBL *P. aeruginosa* strains: PA01, PA02, and PA03. The evidence suggests that a bacteriocin-like molecule of around 55 kDa was responsible for the inhibitory activity. The gene for enterocin, one of the two bacteriocin structural genes found in *E. faecium* KAE01, is thought to be the most plausible candidate to have produced this peptide because it has been shown to produce cyclic peptides and to be able to form oligomers. These properties could explain the protease resistance, and oligomer aggregation could have produced the broad peptide band. However, efforts are being made to chemically extract and purify this inhibitory substance that resembles bacteriocin.

The evaluation of the three-dimensional (3D) structure of KAE01 and the matrix protein via the Ramachandran plot and qmean factor revealed that the models are in good agreement with the experimental structures (Figure 7, Figure 8 and Figure 9). Two predicted models of KAE01 EntP that were obtained with different methods (AB and RoseTTa fold) on the Robetta server show completely different 3D structures. A Ramachandran plot analysis was performed to assess the quality of the predicted structure. Most residues (97.91% and 100%) of the KAE01 EntP structure predicted with the RoseTTa fold method and AB method were grouped in the most favored region, whereas only 2.08% and 0% of the residues lay in the outlier region, which indicates good structure quality. The qmean factors of KAE01 EntP predicted with the RoseTTa fold method and AB method are −1.10 and −0.95, respectively. A qmean z-score around zero confirmed that the model has good agreement with the experimental structures. According to the evaluated parameters predicted by the models for KAE01 EntP, the model of KAE01 EntP predicted by the AB method of the Robetta server is better. The predicted models of KAE01 EntA that were obtained with different methods (AB and RoseTTa fold) on the Robetta server have the same 3D structure. A Ramachandran plot analysis was performed to assess the quality of the predicted structure. All residues (100%) of the predicted KAE01 EntP structure were grouped in the most favored region, which indicates good structure quality. The qmean factor of predicted KAE01 EntA is 0.40. A qmean z-score around zero confirmed that the model has good agreement with the experimental structures. The two bacteriocins, KAE01 EntP and KAE01 EntA, were docked to the matrix protein of *P. aeruginosa* to predict their best binding modes in the complex (Table 5).

**Figure 12:**
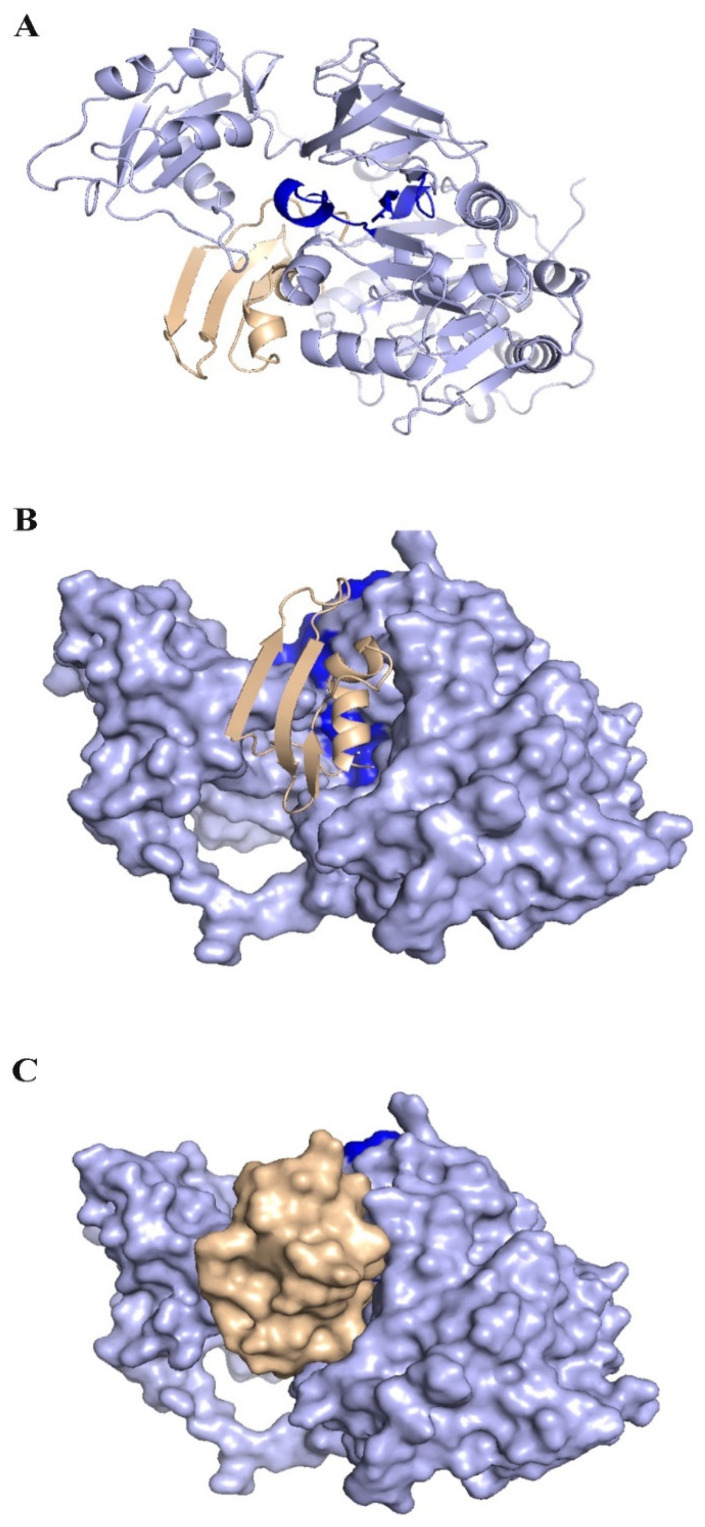
KAE01 EntP complexed with matrix protein. KAE01 EntP model (wheat color) with AB method complexed with matrix protein (light-blue color). Highly conserved residues Gly307, Gly302, Ala278, Gly279, His308, and Tyr211 in N-terminal domain are highlighted in dark blue. (**A**) Shows Ribbon representations (**B**) KAE01 Cavity occupied surface of protein (**C**) Both are represented in surfaces.

KAE01 EntP was located in the valley between two domains of the matrix protein (G279, S280, P281, L282, A256, and P255 in the N-terminal domain and N489, D490, N491, G492, and K493 in a loop of the C-terminal domain) (Figure 12).

**Figure 13:**
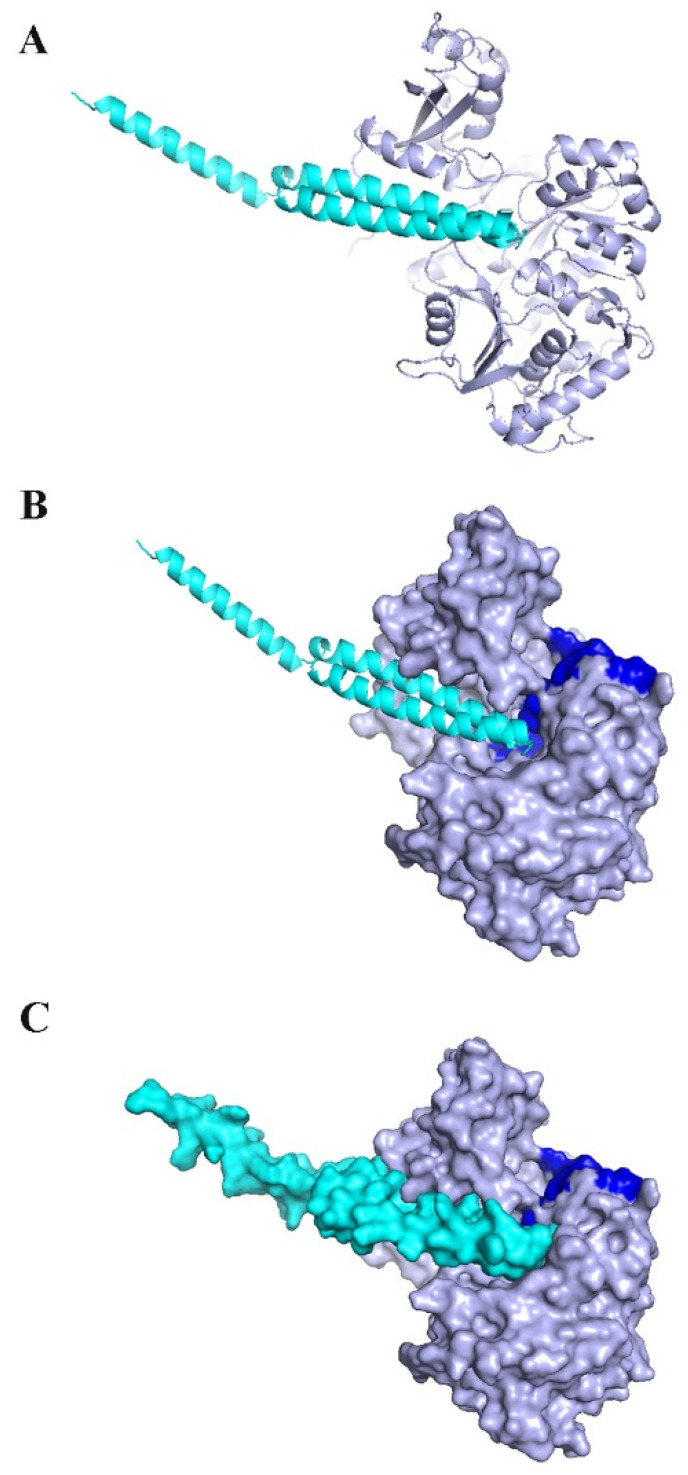
KAE01 EntA complexed with matrix protein. KAE01 EntA (cyan color) complexed with matrix protein (light-blue color). Highly conserved residues Gly307, Gly302, Ala278, Gly279, His308, and Tyr211 in N-terminal domain are highlighted in dark blue. (**A**) Shows Ribbon representations (**B**) KAE01 Cavity occupied surface of protein (**C**) Both are represented in surfaces.

The KAE01 EntA bacteriocin binds to the matrix protein of *P. aeruginosa* in three clusters with 46 members from 1000 bootstraps in the best binding mode with a binding energy of −12.4 Kcal/mol and an inhibition constant of 0.8 nM (Table 5). KAE01 EntA localizes near the conserved region of the N-terminal domain of the matrix protein of *P. aeruginosa* (G166, S167, and T169 loop in the N-terminal domain and F208 F209, G210, G279, and Ser280 residues in the N-terminal domain) (Figure 13). Since the mode of binding of EntA and EntP was found to be maintained by amino acid residues other than those in the active site of the protein, the binding of both peptides involved different types of interactions, such as hydrogen bonding, *π–π*, and hydrophobic interactions. Therefore, the results suggest a vigorous mode of binding that could induce allosteric communication in the case of both peptides. 

## 5. Conclusions

This study suggests that enterocins produced by *E. faecium* have promising antibacterial capabilities against the MDR strain of *P. aeruginosa*. Based on docking results, it is concluded that KAE01 EntP, in comparison with KAE01 EntA, showed better inhibitory effects on the matrix protein of *P. aeruginosa*. These findings can lead to further studies to develop bacteriocin-based antibacterial drugs. The stability of the bacteriocin at various pH values and temperatures and in the presence of enzymes broadens the scope of its extensive usage in the medical, agri-food, and pharmaceutical sectors. Moreover, the use of in silico methods in bacteriocin research has significantly increased in recent times. The two most renowned web-based databases now provide computational tools for mining bacteriocin genomes, similarity searches, alignments, physicochemical investigation, Markov simulations, and structure predictions. Therefore, it is obvious that bacteriocin research will continue to advance quickly, and its future will be decided by the development of cutting-edge technologies or computational techniques.

## Figures and Tables

**Table 1:** Antimicrobial activity of purified enterocin KAE01 against *P. aeruginosa* strains.

Enterococcus	Indicator Strain	ZOI (mm)	*p*-Value
KAE 01	PA 01	12 ± 0.1	0.001 *
	PA 02	11 ± 0.2	
	PA 03	12 ± 0.5	

(Zones of inhibition (ZOIs): 0–10 mm: resistant; 10.1–16 mm: intermediate zones; 17+ mm: sensitive). * Significant differences at *p* < 0.05.

**Table 2:** Inhibitory effects of enterocin at various pH values against PA01.

pH Value	ZOI (mm)	*p*-Value
2.0	17.9 ± 0.9	0.001 *
3.0	18.8 ± 0.1	
4.0	19.5 ± 2	
5.0	23.2 ± 0.1	
6.0	22.4 ± 0.1	
7.0	20.8 ± 0.2	
8.0	17.9 ± 0.1	
9.0	16.2 ± 0.5	
10.0	15.7 ± 0.6	

(Zones of inhibition (ZOIs): 0–10 mm: resistant; 10.1–16 mm: intermediate zones; 17+ mm: sensitive). * Significant differences at *p* < 0.05.

**Table 3:** Inhibitory effects of enterocin after various temperatures against PA01.

Temperature (°C)	ZOI (mm)	*p*-Value
−20	23.8 ± 0.1	0.001 *
4.0	24.0 ± 0.1	
30	24.5 ± 0.05	
60	22.8 ± 0.1	
90	21.1 ± 0.3	
100	20.8 ± 0.6	
121 *	17.4 ± 0.5	

(Zones of inhibition (ZOI): 0–10 mm: resistant; 10.1–16 mm: intermediate zones; 17+ mm: sensitive). * Significant differences at *p* < 0.05.

**Table 4:** Inhibitory effects of enterocin in presence of proteolytic enzymes against PA01.

Proteolytic Enzymes	ZOI (mm)	*p*-value
Pepsin	5.5 ± 0.1	0.001 *
Trypsin	8.6 ± 0.6	
Proteinase K	10.5 ± 0.2	
Alkaline Protease	7.5 ± 0.2	

(Zones of inhibition (ZOIs): 0–10 mm: resistant; 10.1–16 mm: intermediate zones; 17+ mm: sensitive). * Significant differences at *p* < 0.05.

**Table 5:** Weighted scores, binding affinity (ΔG), and dissociation constant (Kd) of the interaction of each peptide with the matrix protein of *P. aeruginosa*.

Peptides	Cluster	Members (Docked Conformations)	Representative	Weighted Score (KJ/mol)	ΔG (Kcal/mol)	Kd (M) at 25 °C
KAE01 EntA	3	46	Center Lowest-energy structure	−955.8 −1046.0	−12.4	8 × 10^−10^
KAE01 EntP	2	49	Center Lowest-energy structure	−732.4 −732.4	−16.4	8.9 × 10^−13^

## Data Availability

Not applicable.
